# The effect of heat waves on mortality in susceptible groups: a cohort study of a mediterranean and a northern European City

**DOI:** 10.1186/s12940-015-0012-0

**Published:** 2015-03-29

**Authors:** Daniel Oudin Åström, Patrizia Schifano, Federica Asta, Adele Lallo, Paola Michelozzi, Joacim Rocklöv, Bertil Forsberg

**Affiliations:** Department of Public Health and Clinical Medicine, Division of Occupational and Environmental Medicine, Umeå University, Umeå, Sweden; Department of Epidemiology, Lazio Regional Health Service, Rome, Italy; Department of Public Health and Clinical Medicine, Division of Epidemiology and Global Health, Umeå University, Umeå, Sweden

**Keywords:** Heat wave, Mortality, Susceptible groups

## Abstract

**Background:**

Climate change is projected to increase the number and intensity of extreme weather events, for example heat waves. Heat waves have adverse health effects, especially for the elderly, since chronic diseases are more frequent in that group than in the population overall. The aim of the study was to investigate mortality during heat waves in an adult population aged 50 years or over, as well as in susceptible subgroups of that population in Rome and Stockholm during the summer periods from 2000 to 2008.

**Methods:**

We collected daily number of deaths occurring between 15th May and 15th September each year for the population above 50 as well as the susceptible subgroups. Heat wave days were defined as two or more days exceeding the city specific 95th percentile of maximum apparent temperature (MAT). The relationship between heat waves and all-cause non-accidental mortality was investigated through time series modelling, adjusting for time trends.

**Results:**

The percent increase in daily mortality during heat waves as compared to normal summer days was, in the 50+ population, 22% (95% Confidence Interval (CI): 18-26%) in Rome and 8% (95% CI: 3-12%) in Stockholm. Subgroup specific increase in mortality in Rome ranged from 7% (95% CI:–17-39%) among survivors of myocardial infarction to 25% in the COPD (95% CI:9-43%) and diabetes (95% CI:14-37%) subgroups. In Stockholm the range was from 10% (95% CI: 2-19%) for congestive heart failure to 33% (95% CI: 10-61%) for the psychiatric subgroup.

**Conclusions:**

Mortality during heat waves increased in both Rome and Stockholm for the 50+ population as well as in the considered subgroups. It should be evaluated if protective measures should be directed towards susceptible groups, rather than the population as a whole.

**Electronic supplementary material:**

The online version of this article (doi:10.1186/s12940-015-0012-0) contains supplementary material, which is available to authorized users.

## Background

The on-going climate change is predicted to result in a growing number of extreme meteorological events throughout Europe [1]. In particular, the effect of high temperature and heat waves is already having an important impact on public health in terms of increased mortality [2-5]. In developed countries, increasing longevity is steadily increasing the proportion of elderly persons, one of the population groups most vulnerable to heat, both in terms of mortality and morbidity [6-8].

An increased risk of dying on extremely hot days has been reported among individuals with heart disease as well as other cardiovascular conditions such as congestive heart failure, conduction disorders, and cerebrovascular disease [7,9-15], and a recent study reported that extreme heat increases the risk of dying after an acute myocardial infarction (MI) [16]. Other epidemiological studies have shown an increased vulnerability to elevated temperatures among those with respiratory diseases [11,14,17], diabetes [9,13,17], and psychiatric disorders [10,11,14,18,19].

Increased susceptibility to heat waves in older individuals might be due to impaired thermoregulation responses or reduced ability for an individual to protect him or herself from heat stress [12,18]. Thermoregulatory responses to heat stress are impaired in individuals with cardiovascular disease, and these individuals are more vulnerable to heat-induced increases in blood viscosity, red blood cell number, cholesterol levels, and platelet counts that can lead to coronary and cerebral thrombosis [20,21]. In patients with diabetes, the skin blood flow response is altered and metabolic alterations associated with the disease can further reduce heat tolerance [21]. Thermoregulation can be impaired also in people with chronic respiratory diseases for whom any respiratory adjustment (i.e. for dissipating excess heat from respiration and for compensating the greater cardiovascular workload) is challenging, often leading to hyperventilation and exacerbations [2,21]. Psychiatric conditions increase susceptibility to heat for both behavioural and physiological reasons; awareness of heat related risks might be reduced and prescribed medications might impair the body’s thermoregulation capacity [21].

When considering all of the available evidence, it becomes clear that the short-term effect of heat in cohorts characterized by specific clinical susceptibility factors has not yet been fully investigated. Quantifying the effect of heat on specific subgroups might be of interest to public health authorities. The effect of heat on susceptible individuals might differ in different geographical areas according to climatic conditions, diagnostic principles, health care facilities, and other local characteristics.

In the current study, we used data from Rome, Italy, and Stockholm, Sweden, which represent very different climatic conditions, the first being typical of the Mediterranean zone and the second of the North Continental zone. Consequently a heat wave in Rome will be defined, in absolute terms, differently from a heat wave in Stockholm. We evaluated the effect of heat waves on mortality in five subgroups of susceptible, chronically ill individuals aged 50 years and older diagnosed with congestive heart failure (CHF), chronic obstructive pulmonary disease (COPD), diabetes, or psychiatric disorders as well as survivors of MI in the two cities. We compared these effects to those in the general population and to the subgroup of the general population not belonging to any of the defined subgroups (the Low Risk subgroup, LR). We also evaluated temporal and geographic variations as well as effect modification by age and gender.

## Methods

We used an open (once a year) cohort approach covering the time period 2000–2008 and collected daily all-cause mortality data for the susceptible subgroups as well as the LR subgroup and the general population. Inclusion in the general population was open once a year, and to be included in the study population the individual had to be a resident in either of the two cities and had to be 50 years or older on the 15th of May each year. The general population aged 50 years and older was retrieved from the population registries of both cities and updated annually from May 2000. The susceptible subgroups were chosen based on a literature search and defined prior to analyses. An individual belonging to the general population entered a susceptible subgroup at the date of his or her first hospitalization for their diagnosis (at any time of year), or 15th of May for prevalent cases, and stayed in the cohort until death, migration from the study area, or the end of the study. For each hospitalization, we checked the hospital registration system back to 1997 to verify that there were no previous hospitalizations for the same diagnosis for the same individual. For survivors of MI, we included individuals who were discharged alive after a diagnosis of MI at least 28 days but not more than three years from the 15th of May of the year of enrolment. All subgroups were updated with new entries annually on the 15th of May. During the period between the 15th of May and the 15th of September of each year, all investigated groups were studied with regards to the daily number of deaths occurring in each of the groups. The ICD codes used for defining the susceptible subgroups are presented in Additional file 1.

Hospitalization data were extracted from The Regional Hospital Information System in the Lazio region (including Rome) and from the patient register at The National Board of Health and Welfare for the City of Stockholm.

In order to investigate effect modification by age and sex, we stratified the analyses accordingly. We divided all investigated groups into those aged 50–74 years and those aged 75 years and older.

We first investigated associations between high temperature and mortality using daily measures of mean, maximum, minimum, and maximum apparent temperature (MAT). Ambient temperature likely differs from skin temperature, which is what signals the body to start thermoregulation [22]. Therefore, incorporating humidity into the apparent temperature may be more appropriate. Apparent temperature (AT) is calculated from the daily mean temperature (T) and the daily mean dew point temperature (DT) with the following formula: AT=−2.653 + 0.994*T + 0.0153*(DT) [23]. Measurements included the 90th, 95th, 98th, and 99th percentiles of daily temperature observations based on measurements of the summer months in 1995–2008. Using Poisson regression models we ranked the Akaike Information Criteria’s for all temperature measurements and chose the metric and percentile with the lowest combined rank for all groups, which were MAT and its 95th percentile.

We then defined a heat wave as two consecutive days with temperatures exceeding the 95th percentile of the MAT. To allow for a delayed effect of heat, we also considered the two days following a heat wave day to be heat wave days. To calculate the cut-off percentile, we used daily temperature observations for the summer months from the period 1995–2008. Data for Rome were obtained from the airport station closest to the city and for Stockholm from the station at Bromma City airport.

### Statistical methods

The analysis of the relationship between heat waves and mortality during the period 2000–2008 assumed that the daily counts of mortality followed a Poisson distribution (we found no evidence of overdispersion). A Generalised Additive Model was fitted to the data.

The yearly time trends that occurred throughout the study period were described by a cubic spline with a fixed 4 degrees of freedom for each year (doy). The doy variable is a count variable taking a value of 1 on the 15th of May, 2 on the 16th of May, etc. on a yearly basis. In order to account for increasing size of the cohorts over time we included the number of individuals included in each cohort as an offset in the models. The day of the week and national holidays were included as categorical and binary variables, respectively, yielding the final model:

$$ \begin{array}{l} Mortalit{y}_t\sim Poisson\left({\mu}_t\right)\\ {} log\left({\mu}_t\right) = intercept + weekday + holiday + S\left(doy,\ df = 4\  per\  year\right) + heat\  wave\end{array} $$

In order to ascertain differences between age and sex and between the susceptible subgroups and the LR subgroup, we calculated the Relative Risk Ratio (RRR) as the ratio of the RR of each investigated group to the RR of the reference subgroup. We evaluated statistical significance based on the calculated Z-score and corresponding p-value [24].

To determine any linear trends over time, we investigated the yearly estimates of the RR of mortality by including an interaction term between year and heat wave in the model. We also investigated the period before (2000–2002) and after (2005–2008) the European heat wave of 2003 in order to investigate if any changes in vulnerability could be detected following such an extreme event.

The SAS version 9.2 software package was used to create datasets and variables. R version 2.13.1 was used for the statistical models and the creation of outputs. The R code used is available in Additional file 2.

## Results

Descriptive statistics for the general and LR population above age 50 and for each of the investigated susceptible subgroups for the two cities are presented in Table 1. Rome and Stockholm have an approximate number of residents of 2.7 million and 1.3 million, respectively. The percentage of the overall population aged 50 years or older is approximately 42% in Rome and 40% in Stockholm.

**Table 1:** **Descriptive statistics of the investigated groups in Rome and Stockholm, 2000-2008**

		**Average size of group 2000-2008**		**Mortality (daily number of deaths)**				
	Group	Average size	% Women	N total	Mean	Median	Min	Max
Rome	CHF	17,668	49.9	6,820	6.1	6	0	20
	COPD	17,235	46.8	4,150	3.7	4	0	12
	DIABETES	49,233	48.5	8,719	7.8	8	0	20
	MI	7,045	30.9	1,190	1.1	1	0	6
	PSYCHIATRIC	22,837	68.1	4,437	4.0	4	0	14
	LR	1,011,766	56.6	47,180	42.6	41	20	89
	TOTAL	1,106,511	56.2	68,753	61.6	60	33	124
	Group	Mean	% women	N total	Mean	Median	Min	Max
Stockholm	CHF	20,463	54.3	12,324	11.0	11	0	24
	COPD	3,811	58.6	2,370	2.1	2	0	8
	DIABETES	16,955	46.1	5,636	5.1	5	0	13
	MI	5,591	40.0	1,813	1.6	1	0	7
	PSYCHIATRIC	11,012	43.8	1,712	1.5	1	0	8
	LR	465,232	55.1	24,157	21.8	22	8	39
	TOTAL	512,964	54.5	41,778	37.7	38	21	63

CHF**-**Congestive Heart Failure.

COPD**-**Chronic Obstructive Pulmonary Disease.

MI**-**Survivors to Myocardial Infarction.

LR**-**Low Risk Subgroup.

The MAT in Rome ranged between 15.9°C and 40.2°C (with a mean of 29.5°C and a standard deviation (SD) of 4.5°C), and the MAT in Stockholm ranged between 3.1°C and 31.1°C (with a mean of 18.6°C and a SD of 4.7°C). The 95th percentile for MAT was 35.9°C in Rome and 26.0°C in Stockholm. The total number of heat wave days in Rome were 57, ranging between 0 in 2004 to 16 in 2006. In Stockholm the total number of heat wave days were 61, ranging from 0 in 2000 to 13 in 2006.

**Figure 1:**
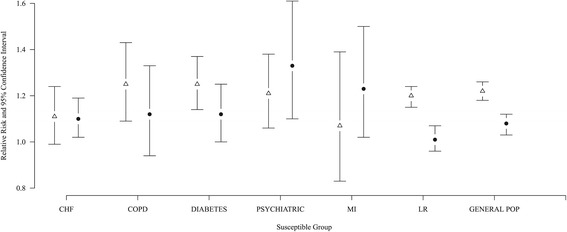
**Heat Wave related mortality for susceptible groups.** Open triangle: Rome; Filled circle: Stockholm.

Figure 1 shows the RR and 95% confidence interval (CI) of mortality associated with heat wave days compared with non-heat wave days by city for each of the investigated groups. Mortality was increased in both cities during heat waves compared to non-heat wave days for all investigated groups, although the increase was not statistically significant for the MI subgroup in Rome or the COPD and NSC subgroups in Stockholm. The RR and 95% CI of dying during a heat wave ranged from 1.07 (0.83–1.39) for the MI subgroup to 1.25 (1.09–1.43) for the COPD subgroup and 1.25 (1.14-1.37) for the diabetes subgroup in Rome. We found no evidence to suggest that the increase in mortality among the susceptible subgroups differed significantly from the LR subgroup in Rome. In Stockholm the RR and 95% CI of dying during a heat wave ranged from 1.01 (0.96–1.07) for the LR subgroup to 1.33 (1.10–1.61) for the psychiatric subgroup. In Stockholm we found that the psychiatric subgroup differed significantly from the LR subgroup. Furthermore we observed borderline significant differences between the LR subgroup and CHF, diabetes, and survivors of MI subgroups as well as the total population.

Table 2 presents the RRs for the total population as well as stratified for sex and age. Age was found to modify the effect in Rome for the general population only, and for this group the RRR was significantly higher for the 75+ age group than for the 50–74 age group. Borderline significant differences between age groups were observed in the diabetes and LR subgroups in Rome. In Stockholm, age was not found to modify the effect in any investigated group.

**Table 2:** **Relative Risks (RR) and Relative Risk Ratios (RRR) for the different groups**

	**Group**		**Total**	**Men**	**Women**	**50-74 years**	**75+ years**
Rome	CHF	RR (95% CI)	1.11 (0.99–1.24)	1.10 (0.93–1.29)	1.16 (1.00–1.36)	0.96 (0.75–1.25)	1.18 (1.04–1.33)
		RRR (95% CI)		1.00	1.06 (0.85–1.33)	1.00	1.22 (0.92–1.61)
	COPD	RR (95% CI)	1.25 (1.09–1.43)	1.25 (1.05–1.49)	1.25 (1.02–1.55)	1.33 (1.03–1.73)	1.22 (1.05–1.43)
		RRR (95% CI)		1.00	1.00 (0.76–1.32)	1.00	0.92 (0.68–1.24)
	DIABETES	RR (95% CI)	1.25 (1.14–1.37)	1.10 (0.96–1.26)	1.42 (1.25–1.62)	1.10 (0.93–1.31)	1.33 (1.19–1.48)
		RRR (95% CI)		1.00	1.30 (1.07–1.57)	1.00	1.20 (0.98–1.48)
	PSYCHIATRIC	RR (95% CI)	1.21 (1.06–1.38)	1.11 (0.89–1.37)	1.28 (1.09–1.51)	1.17 (0.90–1.52)	1.23 (1.05–1.42)
		RRR (95% CI)		1.00	1.16 (0.89–1.52)	1.00	1.05 (0.78–1.40)
	MI	RR (95% CI)	1.07 (0.83–1.39)	0.97 (0.68–1.44)	1.23 (0.82–1.83)	1.12 (0.70–1.80)	1.04 (0.75–1.45)
		RRR (95% CI)		1.00	1.27 (0.74–2.19)	1.00	0.93 (0.52–1.65)
	LR	RR (95% CI)	1.20 (1.15–1.24)	1.11 (1.05–1.18)	1.27 (1.21–1.34)	1.13 (1.06–1.21)	1.23 (1.17–1.29)
		RRR (95% CI)		1.00	1.15 (1.06–1.24)	1.00	1.08 (0.99–1.18)
	GENERAL	RR (95% CI)	1.22 (1.18–1.26)	1.13 (1.08–1.19)	1.30 (1.24–1.36)	1.13 (1.06–1.20)	1.26 (1.21–1.31)
		RRR (95% CI)		1.00	1.15 (1.07–1.22)	1.00	1.12 (1.04–1.18)
	Group		Total	Men	Women	50–74 years	75+ years
Stockholm	CHF	RR (95% CI)	1.10 (1.02–1.19)	1.15 (1.03–1.26)	1.06 (0.96–1.18)	1.01 (0.85–1.20)	1.13 (1.04–1.23)
		RRR (95% CI)		1.00	0.92 (0.79–1.08)	1.00	1.12 (0.92–1.35)
	COPD	RR (95% CI)	1.12 (0.94–1.33)	1.35 (1.06–1.73)	0.95 (0.75–1.21)	1.24 (0.94–1.63)	1.05 (0.84–1.31)
		RRR (95% CI)		1.00	0.70 (0.50–0.99)	1.00	0.85 (0.60–1.21)
	DIABETES	RR (95% CI)	1.12 (1.00–1.25)	1.16 (1.00–1.36)	1.08 (0.91–1.26)	1.14 (0.96–1.35)	1.12 (0.97–1.30)
		RRR (95% CI)		1.00	0.92 (0.74–1.15)	1.00	0.98 (0.78–1.23)
	PSYCHIATRIC	RR (95% CI)	1.33 (1.10–1.61)	1.28 (0.99–1.65)	1.40 (1.04–1.88)	1.25 (0.99–1.58)	1.52 (1.06–2.16)
		RRR (95% CI)		1.00	1.09 (0.74–1.61)	1.00	1.21 (0.79–1.85)
	MI	RR (95% CI)	1.23 (1.02–1.50)	1.27 (0.97–1.67)	1.20 (0.91–1.58)	1.18 (0.79–1.78)	1.24 (0.99–1.55)
		RRR (95% CI)		1.00	0.94 (0.64–1.39)	1.00	1.05 (0.66–1.67)
	LR	RR (95% CI)	1.01 (0.96–1.07)	1.03 (0.95–1.12)	1.00 (0.92–1.08)	1.02 (0.92–1.14)	1.03 (0.95–1.10)
		RRR (95% CI)		1.00	0.97 (0.87–1.08)	1.00	1.00 (0.88–1.14)
	GENERAL	RR (95% CI)	1.08 (1.03–1.12)	1.12 (1.05–1.19)	1.04 (0.99–1.11)	1.07 (0.98–1.16)	1.09 (1.04–1.15)
		RRR (95% CI)		1.00	0.93 (0.86–1.03)	1.00	1.02 (0.93–1.13)

CHF-Congestive Heart Failure.

COPD-Chronic Obstructive Pulmonary Disease.

MI-Survivors to Myocardial Infarction.

LR-Low Risk Subgroup.

In Rome, sex was found to modify the effect of heat waves in the diabetes and LR subgroups, as well as for the general population, with women being more vulnerable than men. In Stockholm, sex was found to modify the risk only in the COPD subgroup, with men being more vulnerable than women. No evidence of a trend over time in the yearly city-specific RR estimates was found for either city, except for the CHF subgroup in Stockholm where a statistically significant increasing trend over time was detected (see Additional file 3).

Table 3 presents the results when comparing the time periods 2000–2002 and 2005–2008. In Rome, the point estimates of the RRs were, with the exception of the COPD and diabetes subgroups, lower during the latter period. In Stockholm, the contrary was found and the RRs were higher during the latter period, with the exception of the psychiatric disease and survivors to MI subgroups. Even though no statistical differences between the two periods are found for any of the investigated groups it is a trend worth noticing.

**Table 3:** **Temporal variation in Relative Risks (RR) before and after heat wave of 2003**

**Period**	**CHF**	**COPD**	**Diabetes**	**Psychiatric**	**MI**	**LR**	**Total**
Rome							
2000–02	1.14 (0.92–1.35)	1.15 (0.88–1.50)	1.18 (0.99–1.41)	1.20 (0.93–1.55)	1.23 (0.78–1.96)	1.27 (1.20–1.36)	1.27 (1.21–1.34)
2005–08	1.06 (0.91–1.23)	1.30 (1.10–1.53)	1.22 (1.08–1.39)	1.06 (0.89–1.27)	0.87 (0.59–1.29)	1.15 (1.08–1.22)	1.19 (1.14–1.25)
Stockholm							
2000–02	1.01 (0.88–1.15)	0.97 (0.71–1.33)	0.98 (0.80–1.20)	1.40 (0.98–2.01)	1.19 (0.85–1.68)	1.06 (0.97–1.17)	1.08 (1.00–1.16)
2005–08	1.16 (1.05–1.28)	1.22 (0.98–1.53)	1.21 (1.05–1.40)	1.15 (0.90–1.48)	1.30 (1.00–1.68)	1.05 (0.97–1.13)	1.11 (1.05–1.18)

CHF-Congestive Heart Failure.

COPD-Chronic Obstructive Pulmonary Disease.

MI-Survivors to Myocardial Infarction.

LR-Low Risk Subgroup.

## Discussion

We selected subgroups that were assumed to be more susceptible to heat wave exposure and investigated how these subgroups–as well as the LR and the general population aged 50 years and older–are affected by heat waves in Rome and Stockholm. In both cities, we found increased risk of mortality among the susceptible groups, as well as the LR subgroup and general population during heat waves compared to non-heat wave days. In Rome, all groups except the CHF and survivors to MI subgroups had significantly elevated risks of mortality. The size of the MI subgroup in Rome is quite small however, resulting in low statistical power to detect differences. In Rome the investigated subgroups with pre-existing disease do not appear to be more susceptible than the general population or the LR subgroup. In Rome the excess of risk experienced by the chronic ill cohorts didn’t seem to differ from that of the LR subgroup or from the general population. This is in line with a previous study in which it was shown that chronic illnesses were not effect modifiers for the risk of dying during heat waves with the exception of COPD in those aged 65–74 years [14] Among older people (75 +) instead sociodemographic factors as living alone and increasing age were the main effect modifiers [14]. In that study to have chronic illness among the oldest (75+) was instead associated with a lower RR of dying during heat waves episode. This was interpreted by the authors as the consequence of a higher level of medical attention on those subjects with potentially extra vigilance.

In Stockholm we found increased risk of dying during heat waves among all investigated groups. With exception from the COPD and LR subgroups, these increases were found to be statistically significant. The small size of the COPD subgroup in Stockholm provided low statistical power to detect differences. We found that the psychiatric subgroup differed significantly from the LR subgroup and borderline significant differences between the LR subgroup and the CHF, diabetes, survivors of MI subgroups as well as the general population were observed. With a point estimate for the LR subgroup consistently below the other investigated groups, we may have identified some of the groups driving increased mortality during heat waves in Sweden. Higher RRs associated with heat waves have been reported for individuals hospitalised for MI (among those aged 65 years and older), psychiatric disorders, and COPD (among those aged less than 65 years) [25]. However, the previous study did not use the same reference group according to the cohort approach, and the results of that study are difficult to compare to the results of the current study.

In this study we found no strong overall evidence that age is an effect modifier for mortality risk in either city, except for the general population in Rome. This in line with a previous study in Italy where the population aged 75 years or older had generally higher RRs of dying during a heat wave compared to the those aged 65–74 years, although the results of that study were not statistically significant [14]. However increasing age in the 75 and over age group was a significant effect modifier of the susceptibility to heat. A recent study in the US found that mortality rates from a 10°F increase in same-day temperatures among the elderly have become more similar to those of the younger population over time [26].

Interestingly we found contrasting patterns of sex in the two cities. In the groups where we found effect modification by sex, women were more susceptible than men in Rome but in Stockholm men were more susceptible than women. The results for Rome are in line with previous findings [14]. In Stockholm, we found, significant differences between men and women in the COPD subgroup, suggesting that men may be more vulnerable than women in this group. Previously, no modification by sex in the general population was reported for the period 2000–2009 [27], however, the current study only focused on the population aged 50 years and older so the results of the two studies are not entirely comparable.

The yearly estimates of mortality did not indicate a trend in vulnerability among any of the investigated groups in either Rome or Stockholm. However, when comparing the periods 2000–2002 and 2005–2008 we found a tendency for lower estimates during the latter period for most groups in Rome and higher estimates for most groups in Stockholm, although none of the differences reached statistical significance. These findings for Rome are in line with the on-going work of identifying heat-susceptible groups among the elderly and was already observed on the total elder population in a previous work [28,29]. In Stockholm, and in Sweden in general, public awareness about the negative impacts of elevated temperatures and extreme heat is still relatively low and a heat wave warning system was only introduced after the data collection period in this work. Our findings of increased susceptibility among certain subgroups along with the experiences and good practise during heat waves in Rome might help improve the not only the heat warning system and related advice but also medical practises during heat waves and adaptation of care homes and other buildings in Stockholm and elsewhere.

It is recognised that susceptibility factors might be different in different population. The investigation of these factors among adult and elderly populations is crucial to be able to accurately target prevention activities to alleviate the negative health effects of extreme heat. The potential use of susceptibility factors for this purpose is demonstrated by the heat susceptibility indicator that has been developed in Italy to identify elderly populations at risk during heat waves and that is currently being used for local prevention activities [28]. An action that can be taken in order to mitigate the negative effects of heat is to inform those deemed to be the most susceptible about the dangers of elevated temperatures.

Comparing a city located in the Mediterranean region and a city located in northern Europe advances our understanding of the need for local knowledge about health care systems and about specific groups that are the most vulnerable in a particular region. Even though the literature suggests that certain groups might be more vulnerable during heat waves, it is important to identify these groups through country-specific and region-specific epidemiological studies. A key finding in our study is the large difference in mortality during heat waves between survivors of MI and COPD in Rome and Stockholm, and this suggests that regional estimates are needed to better identify susceptible cohorts. Another key finding is that there seems to be no difference in the risk of dying during heat waves between the different susceptible cohorts and the rest of the population of the same age in Rome. It is possible that this is an expression of the local authorities’ on-going work to identify vulnerable groups and to reduce mortality among these groups to a level that is comparable to the less susceptible group. This raises the question of what can be done in order to reach individuals in Rome who still might be at higher risk but who do not have a specific diagnosis to indicate vulnerability.

### Limitations

Temperature data were collected from two stations assumed to represent exposure throughout the two cities, thus there may be exposure misclassification [30]. Assuming the same exposure throughout an entire city based on a single measurement location could lead to over-or underestimation of the risk of dying during heat waves. Hondula et al. [31] found changing death rates within Philadelphia County in the US suggesting that identification of high-risk areas within a city is important from a public health perspective [31]. The same exposure to extreme temperature might differ at an individual level throughout the population due to the different adaptive measures that are taken to mitigate the effects of the heat. Indoor temperatures seem to be more accurately associated with heat perception than outdoor temperatures, thus the subjective feeling of heat strain might not be accurately reflected by the measured temperature. Furthermore, indoor and outdoor temperatures are poorly correlated due to a number of modifying factors [32]. However, the choice of temperature metric used in our study, the MAT, should not impact the results for the different groups to a great extent. Barnett et al. [33] found strong correlations between different temperature metrics and concluded that, on average, they were equally well suited for studies such as the one presented here [33]. The present study did not attempt to determine the association between temperature increases and heat wave duration, but instead presented estimates that are influenced by both temperature duration and intensity. Increasing risks for susceptible groups during longer heat waves compared to shorter heat waves have been reported previously in Sweden [25]. The effect of temperature on mortality was expected to be small but to provide enough statistical power to detect small associations, we chose to look particularly at those conditions with a higher prevalence in the population.

We used the same metric and percentile cut-off for all groups to compare a Mediterranean city with a northern European city and to compare different cohorts within the cities. However, when studying susceptible subgroups within the population, one must consider potential impairments in regulating body temperatures during heat waves [21]. For certain susceptible groups, it might be more important to be able to cool down during night time thus indicating that minimum temperature might be a better measure of temperature-related mortality. Future studies should seek to identify the best indicator, be it metric or percentile, for temperature-related mortality not only on a regional level, but also on a susceptible group level. We identified our susceptible subgroups based on hospitalizations, but in reality some of the subgroups are likely to be larger and to include individuals who were not hospitalized for their illness. These persons may be less fragile, but also less used to seek health care, why their risk of dying during a heat wave could also be higher.

## Conclusions

We reported increased heat related mortality among the general 50+ population in two cites with very different climatic conditions and identified subgroups of the general population with heightened susceptibility during heat waves in Rome and Stockholm. The increased risk in our results can be used to improve the on-going work with heat warning systems by incorporating regional estimates when targeting susceptible individuals.

## Additional files

Additional file 1:
**ICD CODES.**
Additional file 2:
**R-code.**
Additional file 3:
**Yearly estimates of Relative Risks (RR) 2000–2008.**

## Abbreviations

CHF: Congestive heart failure
CI: Confidence interval
COPD: Chronic obstructive pulmonary disease
ICD: International Classification of Disease
LR: Low risk subgroup
MAT: Maximum apparent temperature
MI: Myocardial infarction
RR: Relative risk
RRR: Relative risk ratio

## References

[1] Field CB (2012). Managing the risks of extreme events and disasters to advance climate change adaptation: special report of the intergovernmental panel on climate change.

[2] Michelozzi P, Accetta G, De Sario M, D’Ippoliti D, Marino C, Baccini M (2009). High temperature and hospitalizations for cardiovascular and respiratory causes in 12 European cities. Am J Respir Crit Care Med.

[3] D’Ippoliti D, Michelozzi P, Marino C, De’Donato F, Menne B, Katsouyanni K (2010). Research The impact of heat waves on mortality in 9 European cities: results from the EuroHEAT project. Environ Health.

[4] Baccini M, Kosatsky T, Analitis A, Anderson HR, D’Ovidio M, Menne B (2011). Impact of heat on mortality in 15 European cities: attributable deaths under different weather scenarios. J Epidemiol Community Health.

[5] Rocklov J, Ebi K, Forsberg B (2011). Mortality related to temperature and persistent extreme temperatures: a study of cause-specific and age-stratified mortality. Occup Environ Med.

[6] Sierra F, Hadley E, Suzman R, Hodes R (2009). Prospects for life span extension. Annu Rev Med.

[7] Oudin Åström D, Bertil F, Joacim R (2011). Heat wave impact on morbidity and mortality in the elderly population: a review of recent studies. Maturitas.

[8] Ye X, Wolff R, Yu W, Vaneckova P, Pan X, Tong S (2012). Ambient temperature and morbidity: a review of epidemiological evidence. Environ Health Perspect.

[9] Medina-Ramón M, Schwartz J (2007). Temperature, temperature extremes, and mortality: a study of acclimatisation and effect modification in 50 US cities. Occup Environ Med.

[10] Stafoggia M, Forastiere F, Agostini D, Biggeri A, Bisanti L, Cadum E (2006). Vulnerability to heat-related mortality: a multicity, population-based, case-crossover analysis. Epidemiology.

[11] Stafoggia M, Forastiere F, Agostini D, Caranci N, De’Donato F, Demaria M (2008). Factors affecting in-hospital heat-related mortality: a multi-city case-crossover analysis. J Epidemiol Community Health.

[12] Kovats RS, Hajat S (2008). Heat stress and public health: a critical review. Annu Rev Public Health.

[13] Basu R, Ostro BD (2008). A multicounty analysis identifying the populations vulnerable to mortality associated with high ambient temperature in California. Am J Epidemiol.

[14] Schifano P, Cappai G, De Sario M, Michelozzi P, Marino C, Bargagli AM (2009). Susceptibility to heat wave-related mortality: a follow-up study of a cohort of elderly in Rome. Environ Health.

[15] Basu R (2009). High ambient temperature and mortality: a review of epidemiologic studies from 2001 to 2008. Environ Health.

[16] Madrigano J, Mittleman MA, Baccarelli A, Goldberg R, Melly S, Von Klot S (2013). Temperature, myocardial infarction, and mortality: Effect modification by individual-and area-level characteristics. Epidemiology.

[17] Schwartz J (2005). Who is sensitive to extremes of temperature?: A case-only analysis. Epidemiology.

[18] Bouchama A, Dehbi M, Mohamed G, Matthies F, Shoukri M, Menne B (2007). Prognostic factors in heat wave–related deaths: a meta-analysis. Arch Intern Med.

[19] Zanobetti A, O’Neill MS, Gronlund CJ, Schwartz JD (2013). Susceptibility to mortality in weather extremes: effect modification by personal and small-area characteristics. Epidemiology.

[20] Keatinge WR, Coleshaw SR, Easton JC, Cotter F, Mattock MB, Chelliah R (1986). Increased platelet and red cell counts, blood viscosity, and plasma cholesterol levels during heat stress, and mortality from coronary and cerebral thrombosis. Am J Med.

[21] Kenny GP, Yardley J, Brown C, Sigal RJ, Jay O (2010). Heat stress in older individuals and patients with common chronic diseases. CMAJ.

[22] Ashcroft FM (2002). Life at the Extremes: The Science of Survival.

[23] Kalkstein LS, Valimont KM (1986). An evaluation of summer discomfort in the United State using a relative climatological index. Bull Am Meteorol Soc.

[24] Altman DG, Bland JM (2003). Statistics Notes: interaction revisited: the difference between two estimates. BMJ.

[25] Rocklöv J, Forsberg B, Ebi K, Bellander T (2014). Susceptibility to mortality related to temperature and heat and cold wave duration in the population of Stockholm County. Sweden Glob Health Action.

[26] Bobb J, Peng R, Bell M, Dominici F (2014). Heat-related mortality and adaptation in the United States. Environ Health Perspect.

[27] Åström DO, Forsberg B, Edvinsson S, Rocklöv J (2013). Acute fatal effects of short-lasting extreme temperatures in Stockholm, Sweden: Evidence across a century of change. Epidemiology.

[28] Schifano P, Cappai G, De Sario M, Bargagli A, Michelozzi P (2013). Who should heat prevention plans target? A heat susceptibility indicator in the elderly developed based on administrative data from a cohort study. Healthy Aging Res.

[29] Schifano P, Leone M, De Sario M, De’Donato F, Bargagli AM, D’Ippoliti D (2012). Changes in the effects of heat on mortality among the elderly from 1998–2010: results from a multicenter time series study in Italy. Environ Health.

[30] Armstrong BG (1998). Effect of measurement error on epidemiological studies of environmental and occupational exposures. Occup Environ Med.

[31] Hondula DM, Davis RE, Rocklöv J, Saha MV (2013). A time series approach for evaluating intra-city heat-related mortality. J Epidemiol Community Health.

[32] Franck U, Kruger M, Schwarz N, Grossmann K, Roder S, Schlink U (2013). Heat stress in urban areas: Indoor and outdoor temperatures in different urban structure types and subjectively reported well-being during a heat wave in the city of Leipzig. Meteorol Z.

[33] Barnett AG, Tong S, Clements A (2010). What measure of temperature is the best predictor of mortality?. Environ Res.

