# Supplementary material for: The effect of heat waves on mortality in susceptible groups: a cohort study of a mediterranean and a northern European City
Source: Environ Health. 2015 Mar 29;14:30. doi: 10.1186/s12940-015-0012-0 (PMC4397690; doi:10.1186/s12940-015-0012-0)
Supplement: Additional file 1: — ICD CODES. [file 12940_2015_12_MOESM1_ESM.pdf]

Additional file 1: ICD CODES used for definition of susceptible groups:

Congestive Heart Failure (CHF): (ICD9: 428, ICD10: I50)

Chronic Obstructive Pulmonary Disease (COPD): (ICD9: Main diagnosis: 490–492, 494, 496 or secondary diagnosis 490–492, 494, 496 and main diagnosis 518.5 518.8 786.0 428.0 416.9, ICD10: J43–J44, J47 or secondary J43–J44, J47 and main diagnosis J95.1–I95.3, J96, J98.0, J98.4, J80, R06, I50.9, I27.9)

Diabetes: (ICD9:250, ICD10:E10–14)

Psychiatric disorders: (ICD9: 291–299, 300.4, 301.1, 309.0, 309.1, 311, ICD10: F20–F22, F31, F32, F34, F43)

Survivors to Myocardial Infarction (MI): (ICD9: Main diagnosis 410 or main diagnosis 411, 413, 414, 423.0, 426, 427 (but not 427.5) and secondary diagnosis 410, ICD10: main diagnosis I24, I20, I25 (except I25.2), I31.2, I44, I45, I47, I48, I49, and secondary diagnosis I21)
