# Supplementary material for: The effect of heat waves on mortality in susceptible groups: a cohort study of a mediterranean and a northern European City
Source: Environ Health. 2015 Mar 29;14:30. doi: 10.1186/s12940-015-0012-0 (PMC4397690; doi:10.1186/s12940-015-0012-0)
Supplement: Additional file 2: — R-code. [file 12940_2015_12_MOESM2_ESM.pdf]

Additional file 2. R-code

#Association between heat waves and mortality for the different investigated groups for Rome and Stockholm

```
 Mort_ij <- gam (N_deaths_i ~ as.factor(wday) + as.factor(hday)
                 + s(doy,k=4, by=as.factor(year), fx=T)
                 + hw_tappmax_95 ,
                  data=j, family=poisson, offset=log(N_TotPop_i)    )
```

where i represents the different investigated groups: Congestive Heart Failure (CHF), chronic obstructive pulmonary disease (COPD), diabetes, or psychiatric disorders, survivors of Myocardial Infarction (MI), the Low-Risk subgroup (LR) and the general population.

and j represents data from the cites if Rome and Stockholm

Association between heat waves and mortality for the different investigated groups for Rome and Stockholm before and after the heat wave of 2003

```
j_1 <- subset(j, year %in% 2000:2002)

j_2 <- subset(j, year %in% 2005:2008)

Mort_ij <- gam (N_deaths_i ~ as.factor(wday) + as.factor(hday)
                 + s(doy,k=4, by=as.factor(year), fx=T)
                 + hw_tappmax_95 ,
                  data=j_1, family=poisson, offset=log(N_TotPop_i)    )

Mort_ij <- gam (N_deaths_i ~ as.factor(wday) + as.factor(hday)
                 + s(doy,k=4, by=as.factor(year), fx=T)
                 + hw_tappmax_95 ,
                  data=j_2, family=poisson, offset=log(N_TotPop_i)    )
```

where i represents the different investigated groups: Congestive Heart Failure (CHF), chronic obstructive pulmonary disease (COPD), diabetes, or psychiatric disorders, survivors of Myocardial Infarction (MI), the Low-Risk subgroup (LR) and the general population.

and j represents data from the cites if Rome and Stockholm

Yearly estimates of the impact of heat waves on mortality

```
Year_ij <- gam (N_deaths_i ~ as.factor(wday) + as.factor(hday)
                 + as.factor(year)
                 + s(doy,k=4, by=as.factor(year), fx=T)
                 + hw_tappmax_95:as.factor(year) ,
                  data=j, family=poisson, offset=log(N_TotPop_i)    )
```

where i represents the different investigated groups: Congestive Heart Failure (CHF), chronic obstructive pulmonary disease (COPD), diabetes, or psychiatric disorders, survivors of Myocardial Infarction (MI), the Low-Risk subgroup (LR) and the general population.

and j represents data from the cites if Rome and Stockholm
