# Supplementary material for: The effect of heat waves on mortality in susceptible groups: a cohort study of a mediterranean and a northern European City
Source: Environ Health. 2015 Mar 29;14:30. doi: 10.1186/s12940-015-0012-0 (PMC4397690; doi:10.1186/s12940-015-0012-0)
Supplement: Additional file 3: — Yearly estimates of Relative Risks (RR) 2000–2008. [file 12940_2015_12_MOESM3_ESM.pdf]

Additional file 3. Yearly estimates of Relative Risks (RR) 2000 - 2008

ROME							
**Year**	CHF	COPD	DIABETES	PSYCHIATRIC	MI	LR	TOTAL
**2000**	1.39 (0.96-2.01)	0.91 (0.51-1.62)	1.72 (1.28-2.31)	1.35 (0.82-2.24)	2.58 (1.26-5.28)	1.28 (1.15-1.43)	1.32 (1.21-1.45)
**2001**	0.82 (0.43-1.55)	1.31 (0.69-2.50)	0.91 (0.54-1.53)	0.69 (0.30-1.56)	0.93 (0.22-3.89)	0.99 (0.83-1.18)	0.99 (0.85-1.15)
**2002**	1.14 (0.86-1.52)	1.26 (0.90-1.77)	1.01 (0.79-1.30)	1.31 (0.95-1.81)	0.79 (0.40-1.60)	1.40 (1.28-1.53)	1.35 (1.25-1.46)
**2003**	1.29 (1.00-1.65)	1.20 (0.87-1.66)	1.39 (1.12-1.72)	1.47 (1.13-1.92)	1.40 (0.79-2.48)	1.11 (1.01-1.21)	1.16 (1.07-1.25)
**2004**	NA	NA	NA	NA	NA	NA	NA
**2005**	1.23 (0.87-1.73)	1.16 (0.76-1.77)	1.12 (0.82-1.55)	1.05 (0.68-1.64)	0.61 (0.22-1.66)	1.19 (1.04-1.36)	1.17 (1.05-1.31)
**2006**	0.97 (0.78-1.20)	1.54 (1.24-1.92)	1.30 (1.08-1.55)	1.24 (0.98-1.59)	0.95 (0.55-1.63)	1.16 (1.06-1.26)	1.19 (1.12-1.28)
**2007**	1.22 (0.93-1.59)	1.02 (0.71-1.46)	1.26 (0.99-1.60)	0.94 (0.66-1.34)	1.15 (0.56-2.39)	1.18 (1.05-1.32)	1.17 (1.07-1.28)
**2008**	NA	NA	NA	NA	NA	NA	NA
STOCKHOLM							
**Year**	CHF	COPD	DIABETES	PSYCHIATRIC	MI	LR	TOTAL
**2000**	NA	NA	NA	NA	NA	NA	NA
**2001**	0.96 (0.72-1.26)	1.12 (0.61-2.06)	1.34 (0.93-1.93)	1.24 (0.54-2.82)	1.45 (0.78-2.70)	1.20 (1.01-1.43)	1.16 (1.01-1.33)
**2002**	1.04 (0.89-1.21)	0.87 (0.60-1.28)	0.91 (0.71-1.18)	1.54 (1.01-2.34)	1.20 (0.78-1.86)	1.04 (0.93-1.17)	1.04 (0.95-1.13)
**2003**	1.12 (0.93-1.35)	1.10 (0.72-1.67)	0.95 (0.71-1.28)	1.29 (0.79-2.12)	0.86 (0.52-1.41)	0.95 (0.83-1.08)	1.04 (0.93-1.15)
**2004**	1.18 (0.85-1.63)	0.99 (0.46-2.14)	1.19 (0.73-1.94)	1.84 (0.93-3.61)	1.87 (0.95-3.71)	0.73 (0.55-0.98)	0.95 (0.78-1.15)
**2005**	1.08 (0.86-1.34)	1.15 (0.72-1.83)	1.08 (0.77-1.52)	1.24 (0.73-2.10)	1.34 (0.79-2.28)	1.09 (0.92-1.28)	1.11 (0.98-1.25)
**2006**	1.21 (1.03-1.42)	1.32 (0.93-1.87)	1.17 (0.92-1.49)	0.97 (0.62-1.52)	1.18 (0.76-1.84)	1.10 (0.97-1.24)	1.14 (1.04-1.24)
**2007**	1.22 (0.98-1.53)	1.03 (0.60-1.76)	1.37 (1.02-1.85)	1.57 (0.99-2.49)	1.63 (0.98-2.72)	0.89 (0.74-1.08)	1.06 (0.93-1.21)
**2008**	1.13 (0.89-1.43)	1.06 (0.62-1.80)	1.24 (0.91-1.68)	0.98 (0.55-1.76)	1.13 (0.59-2.16)	1.02 (0.86-1.22)	1.09 (0.95-1.24)

**CHF**	**Congestive Heart Failure**
**COPD**	**Chronic Obstructive Pulmonary Disease**
**MI**	**Survivors to Myocardial Infarction**
**LR**	**Low Risk Subgroup**
